# Association of Health Insurance Literacy With Enrollment in Traditional Medicare, Medicare Advantage, and Plan Characteristics Within Medicare Advantage

**DOI:** 10.1001/jamanetworkopen.2021.46792

**Published:** 2022-02-03

**Authors:** Sungchul Park, Brent A. Langellier, David J. Meyers

**Affiliations:** 1 Department of Health Management and Policy, Dornsife School of Public Health, Drexel University, Philadelphia, Pennsylvania; 2 Department of Health Services, Policy, and Practice, School of Public Health, Brown University, Providence, Rhode Island

## Abstract

**Importance:**

Health insurance literacy helps individuals make informed choices. However, evidence suggests that Medicare beneficiaries experience low health insurance literacy, leading to high-cost or poor-quality coverage choices.

**Objective:**

To examine how health insurance literacy was associated with coverage choices between traditional Medicare (TM) and Medicare Advantage (MA), as well as within MA.

**Design, Setting, and Participants:**

This cross-sectional study included 6627 TM and MA enrollees, using data from the 2015-2016 Medicare Current Beneficiary Survey. Data analyses were conducted between May 1 and June 30, 2021.

**Exposures:**

Three self-reported measures of health insurance literacy (presence of information to make an informed comparison, ease in reviewing and comparing coverage options, and annual review and comparison of coverage options).

**Main Outcomes and Measures:**

Enrollment in TM vs MA and enrollment in an MA plan with different characteristics (star rating, monthly plan premium, in-network maximum out-of-pocket limit, plan type, and provision of supplemental benefits).

**Results:**

We included 6627 Medicare beneficiaries (3578 women [54.0%]; mean [SD] age, 75.13 [7.12] years). A total of 77 individuals were Asian (1.2%), 696 were Black (10.5%), 488 were Hispanic (7.4%), 5277 were non-Hispanic White (79.6%), and 225 (3.4%) were single races not of Hispanic origin (including American Indian or Alaska Native and Native Hawaiian) or were 2 or more races. Medicare Advantage enrollment was higher among individuals with higher health insurance literacy than those with lower health insurance literacy, especially for those who reviewed or compared coverage options annually than among those who did not (38.0%; 95% CI, 36.0%-40.1% vs 27.8%; 95% CI, 25.8%-29.7%). Among MA beneficiaries, those who reviewed or compared coverage options annually were more likely to enroll in plans with 4 to 4.5 stars and plans with monthly premiums of $1 to $50 by 4.6 percentage points (95% CI, 0.1-9.2 percentage points) and 4.8 percentage points (95% CI, 0.6-9.0 percentage points), respectively. However, enrollment in plans with 5 stars was 3.8 percentage points lower (95% CI, –5.8 to –1.9 percentage points) among individuals who reviewed or compared coverage options annually than among those who did not. Among individuals with low socioeconomic status, the likelihood of reviewing or comparing coverage options annually was lower for those with Medicare and Medicaid dual eligibility than for those without it (odds ratio, 0.79; 95% CI, 0.63-0.99).

**Conclusions and Relevance:**

Results of this study suggest that higher health insurance literacy—particularly, annual review and comparison of coverage choices—is associated with higher MA enrollment and choice of a particular MA plan. Policy makers should develop programs to encourage frequent review and comparison of coverage options for informed decision making.

## Key Points

**Question:**

How is health insurance literacy associated with coverage choices between traditional Medicare and Medicare Advantage, as well as within Medicare Advantage?

**Findings:**

In this cross-sectional study of 6627 Medicare beneficiaries, higher health insurance literacy—particularly, annual review and comparison of coverage choices—was associated with higher Medicare Advantage enrollment and choice of low-cost or high-rated Medicare Advantage plans. However, health insurance literacy was low among Medicare beneficiaries with low socioeconomic status.

**Meaning:**

These findings suggest that policy makers should develop programs to make health insurance information accessible and understandable and to encourage annual review and comparison of coverage options, especially for vulnerable populations.

## Teaser

This cross-sectional study of Medicare beneficiaries uses data from the 2015-2016 Medicare Current Beneficiary Survey to examine the association between health insurance literacy and coverage choices between traditional Medicare and Medicare Advantage, as well as within Medicare Advantage.

## Introduction

Choosing health insurance is among the most important financial and health-related decisions a person can make, but this can be challenging owing to the complexity and difficulty of terms and concepts. Thus, navigating health insurance requires considerable health insurance literacy, defined as a person’s ability to seek, obtain, and understand insurance coverage.^1^ Health insurance literacy can help individuals make informed choices that balance their own perceived needs with plan costs and quality. If individuals do not have the information or ability to make an adequate choice, they may make suboptimal choices that do not meet their preference or care needs.

Health insurance literacy is particularly important to Medicare beneficiaries because they can choose among several options to access Medicare coverage. First, they can obtain their Medicare coverage either through the federally administered traditional Medicare (TM) program or the privately run Medicare Advantage (MA) program. Medicare Advantage plans must provide coverage for at least the same services covered by TM, but MA plans can offer additional supplemental benefits^2,3^ and lower prices^4^ than TM. Once beneficiaries have decided to enroll in MA, they are confronted with a choice between multiple plans. Medicare beneficiaries had access to an average of 33 MA plans in 2021.^5^ Medicare Advantage plans vary significantly in terms of costs, coverage, and quality ratings.

Evidence suggests that Medicare beneficiaries choose suboptimal coverage in terms of cost and quality,^6,7,8,9^ which may be attributable to limited health insurance literacy.^10^ Indeed, most Medicare beneficiaries do not use Medicare’s official information sources.^11^ Furthermore, 30% of Medicare beneficiaries reported difficulty understanding the Medicare program,^11^ and 57% did not review or compare coverage options annually.^11^ This low health insurance literacy may lead to impaired decision making, resulting in high-cost or poor-quality coverage choices. Consequently, many Medicare beneficiaries tend to stay in TM or remain in the same MA plan.^12^ However, little is known about the association between health insurance literacy and Medicare beneficiaries’ choice between TM and MA or their choice between different plans offered within MA.

In this study, we examined the association between health insurance literacy and coverage choices between TM and MA as well as within MA. We conducted 4 analyses. In the first analysis, we examined whether enrollment in TM vs MA differed by health insurance literacy. In the second and third analyses, we investigated whether enrollment in an MA plan with a specific type of characteristic varied by health insurance literacy. We also studied what plan-level characteristics were associated with MA plan choice and how this differed by health insurance literacy. Finally, we examined which individual-level factors were associated with high health insurance literacy.

## Methods

### Data and Sample

In this cross-sectional study, we used data from the 2015-2016 Medicare Current Beneficiary Survey, which provides a nationally representative sample of the Medicare population.^13^ The data combine information from Medicare claims and administrative data, with data collected by interview. The survey was conducted via 3 rounds per year. This study was approved by the University of Pennsylvania’s institutional review board and received a waiver of informed consent and HIPAA (Health Insurance Portability and Accountability Act) authorization because the data were deidentified. This study followed the Strengthening the Reporting of Observational Studies in Epidemiology (STROBE) reporting guideline.

We identified Medicare beneficiaries with 12-month continuous enrollment in TM or MA. We excluded individuals who did not have Parts A and B benefits, those who died within the year, and those whose original Medicare eligibility was attributable to end-stage renal disease. In most analyses, the unit of analysis was at the person level. To examine MA plan choice in each MA enrollee’s actual choice set, however, we restructured the data so that the unit of analysis was the plan-person.

### Outcomes

We included 3 types of coverage choice outcomes. The first outcome was a dichotomous indicator of whether the participant enrolled in TM vs any MA plan. For the second and third coverage choice outcomes, we limited analyses to individuals enrolled in an MA plan. The second type of outcome was a series of categorical variables describing the characteristics of the specific plan in which each participant enrolled. We included the following plan characteristics: star rating, monthly plan premium, in-network maximum out-of-pocket limit, plan type, and provision of supplemental benefits. The Centers for Medicare & Medicaid Services (CMS) has implemented a 5-star quality rating system to improve care quality while helping enrollees compare and choose high-quality MA plans. Specifically, star ratings are appraised according to 47 performance measures. The third type of outcome was a dichotomous indicator of whether the participants enrolled in each of the plans in their choice sets. We reshaped the data such that there was 1 observation for every possible MA plan available to each MA enrollee in their county. Thus, the unit of analysis was the plan-person. We included all possible pairings of each enrollee and each plan available in their choice set, from which each enrollee enrolled in exactly 1 plan.

### Independent Variables

Our primary independent variables were 3 self-reported measures of health insurance literacy, which were selected because of their high relevance. Before the enrollment period for TM and MA, participants were asked to answer the following about these variables: “I have the information I need to make an informed comparison among different health insurance choices,” “How easy would you say it is for you to review and compare your Medicare coverage options?” and “How often do you review or compare your Medicare coverage options?” For each measure, respectively, we categorized responses into 2 levels: disagree (completely or somewhat disagree) vs agree (completely or somewhat agree), difficult (very or somewhat difficult) vs easy (very or somewhat easy), and less than annually (never, rarely, or once every few years) vs annually (at least once every year).

To control for differences in patient characteristics, we included the following individual-level covariates: age, sex, race and ethnicity (self-reported), education, income, Medicare and Medicaid dual eligibility, marital status, comorbidities, general health status, number of limitations in activities of daily living, and baseline year. Because differential access to MA plans may lead to differential enrollment, we included county-level MA plan offerings as follows: total number of MA plans overall and the number of MA plans by star rating, plan type, monthly plan premium, and maximum out-of-pocket limit.

### Statistical Analysis

For our analysis using enrollment in TM vs MA as an outcome, we included both TM and MA enrollees. We estimated adjusted rates of MA enrollment across levels of the 3 health insurance literacy measures. For each measure of health insurance literacy, we conducted a linear probability model of MA enrollment while controlling for individual-level characteristics and county-level MA plan offerings described earlier, as well as health insurance literacy. We also included county-fixed associations to additionally adjust for remaining variations in county-level factors. Then, we estimated the adjusted mean values of MA enrollment by health insurance literacy. Prior research has found that MA enrollees tend to be healthier than TM enrollees, suggesting that a direct comparison between TM and MA enrollees is potentially biased.^14^ As a sensitivity analysis, we conducted the analysis described earlier after applying inverse probability of treatment weights.^15^ Specifically, we computed the inverse probability of treatment weights as a propensity for enrolling in MA according to the variables described.

To examine coverage choice within MA, we limited an analysis to individuals who enrolled in an MA plan. For our analyses using MA enrollment by plan characteristics as an outcome, we estimated adjusted rates of MA enrollment by plan characteristics and health insurance literacy. For each measure of health insurance literacy, we conducted multinomial logit regressions of MA enrollment by each plan characteristic while controlling for individual-level characteristics and county-level MA plan offerings described earlier, as well as health insurance literacy. We also included other 4 types of plan characteristics (monthly plan premium, in-network maximum out-of-pocket limit, plan type, and provision of supplemental benefits) to adjust for differences in other plan characteristics. Then, we estimated the adjusted mean values of benefit-specific enrollment in MA by health insurance literacy. We also estimated differences in the adjusted mean values by health insurance literacy.

Because MA plan decisions may be made with several characteristics in mind, we used a conditional logit choice model to account for the simultaneous association of different plan characteristics with plan choice.^16^ For each MA enrollee, we first constructed a choice set that included all plans available in his or her county. Then, using a conditional logit model with a dichotomous outcome of the chosen plan, we estimated the differential associations between an MA plan’s characteristic and the probability that an enrollee would select that MA plan.^17,18^ Our goal was to assess how the influence of MA plan characteristics on plan choice differed by health insurance literacy. Thus, we included plan characteristics described earlier, as well as their interaction terms with health insurance literacy. Then, we estimated the marginal association between a (1-unit or 1-level) change in a plan-level characteristic and the probability of selecting an MA plan across different levels of health insurance literacy.

To examine which individual-level factors were associated with low or high health insurance literacy, we included both TM and MA enrollees. Then, we conducted logistic regression of each measure of health insurance literacy while controlling for individual-level characteristics and county-level MA plan availability described earlier.

Data analyses were conducted between May 1 and June 30, 2021. All *P* values were from 2-sided tests, and results were deemed significant at *P* < .05. All analyses were conducted in Stata version 16.1 (StataCorp LLC).

## Results

We included 6627 Medicare beneficiaries (3578 women [54.0%]; 2933 men [44.3%]; mean [SD] age, 75.13 [7.12] years) (Table 1). A total of 77 individuals were Asian (1.2%), 696 were Black (10.5%), 488 were Hispanic (7.4%), 5277 were non-Hispanic White (79.6%), and 225 (3.4%) were single races not of Hispanic origin (including American Indian or Alaska Native and Native Hawaiian) or were 2 or more races. We found that 4213 Medicare beneficiaries (63.6%) reported having the information to make an informed comparison among insurance choices, and 5356 (80.8%) reported ease in reviewing and comparing coverage options. However, only 3039 Medicare beneficiaries (45.9%) reviewed or compared coverage options annually.

**Table 1.** Sample Characteristics of Traditional Medicare and Medicare Advantage Enrollees

Characteristic	TM enrollees (n = 4803)	MA enrollees (n = 1824)
Weighted No.	29 989 735	13 509 965
Individual-level characteristics of demographic, socioeconomic, and health status		
Age, No. (%), y		
<65	812 (16.9)	190 (10.4)
65-70	720 (15.0)	326 (17.9)
71-75	1001 (20.8)	412 (22.6)
76-80	829 (17.3)	336 (18.4)
>80	1441 (30.0)	560 (30.7)
Men, No. (%)	2243 (46.7)	806 (44.2)
Women, No. (%)	2560 (53.3)	1018 (55.8)
Race and ethnicity, No. (%)		
Hispanic	292 (6.1)	196 (10.7)
Non-Hispanic		
Asian	55 (1.1)	22 (1.2)
Black	505 (10.5)	191 (10.5)
White	3890 (81.0)	1387 (76.0)
Other^a^	160 (3.3)	65 (3.6)
Education, No. (%)		
<High school	688 (14.3)	324 (17.8)
High school completion	1757 (36.6)	738 (40.5)
≥College degree	2344 (48.8)	759 (41.6)
Income, No. (%), $		
<25 000	1895 (39.5)	823 (45.1)
25 000-40 000	1306 (27.2)	556 (30.5)
≥40 000	1602 (33.4)	445 (24.4)
Medicare-Medicaid dual eligibility, No. (%)	780 (16.2)	181 (9.9)
Married, No. (%)	2409 (50.2)	953 (52.2)
Self-reported comorbidity, No. (%)		
Atherosclerosis	510 (10.6)	187 (10.3)
Hypertension	3356 (69.9)	1251 (68.6)
Myocardial infarction	602 (12.5)	227 (12.4)
Stroke	500 (10.4)	198 (10.9)
Coronary heart disease	542 (11.3)	218 (12.0)
Cancer	1838 (38.3)	613 (33.6)
Rheumatoid arthritis	802 (16.7)	285 (15.6)
Diabetes	1529 (31.8)	602 (33.0)
Alzheimer disease and related dementias	165 (3.4)	55 (3.0)
Mental illness	1469 (30.6)	481 (26.4)
General perceived health status, No. (%)		
Good	3785 (78.8)	1483 (81.3)
Poor	1018 (21.2)	341 (18.7)
ADL limitations, No. (%)		
0	2720 (56.6)	1113 (61.0)
1-2	1219 (25.4)	427 (23.4)
≥3	864 (18.0)	284 (15.6)
County-level characteristics of MA plan offering		
Total No. of MA plans, mean (SD)	11.7 (7.4)	14.7 (7.4)
Star rating, mean (SD)		
2-2.5	0.2 (0.6)	0.2 (0.6)
3-3.5	4.6 (4.8)	5.8 (5.4)
4-4.5	6.5 (5.1)	8.2 (5.0)
5	0.4 (1.1)	0.5 (1.1)
Plan type, mean (SD), No. of plans for a given county		
HMO	6.8 (6.4)	9.2 (7.2)
PPO		
Local	3.1 (2.7)	3.4 (2.8)
Regional	1.4 (1.2)	1.4 (1.3)
Other	0.5 (1.8)	0.6 (2.4)
Monthly plan premium, mean (SD), $		
0	3.7 (4.5)	5.5 (5.4)
>0-50	2.8 (2.4)	3.4 (2.8)
>50-100	2.5 (1.6)	2.6 (1.7)
>100	2.7 (2.8)	3.1 (3.0)
Maximum out-of-pocket limit, mean (SD), $		
0-3000	0.5 (1.3)	0.9 (1.8)
>3000-4500	3.8 (4.0)	4.8 (4.0)
>4500-6000	2.1 (1.9)	2.7 (2.1)
>6000	5.3 (4.4)	6.3 (4.7)
Primary independent variables, No. (%)		
I have the information I need to make an informed comparison among different health insurance choices		
Disagree (completely or somewhat disagree)	1786 (37.2)	628 (34.4)
Agree (completely or somewhat agree)	3017 (62.8)	1196 (65.6)
How easy would you say it is for you to review and compare your Medicare coverage options?		
Difficult (very or somewhat difficult)	964 (20.1)	307 (16.8)
Easy (very or somewhat easy)	3839 (79.9)	1517 (83.2)
How often do you review or compare your Medicare coverage options?		
Never, rarely, or once every few years	2782 (57.9)	806 (44.2)
At least once every year	2021 (42.1)	1018 (55.8)

Abbreviations: ADL, activities of daily living; HMO, health maintenance organization; MA, Medicare Advantage; PPO, preferred provider organization; TM, traditional Medicare.

^a^ Other included single races not of Hispanic origin (including American Indian or Alaska Native and Native Hawaiian) or individuals who were 2 or more races.

Medicare beneficiaries with high health insurance literacy were more likely to choose MA than those with low health insurance literacy (Figure). Particularly, 38.0% (95% CI, 36.0%-40.1%) of individuals who reviewed or compared coverage options annually enrolled in MA compared with 27.8% (95% CI, 25.8%-29.7%) for those who did not. There was a similar pattern for the other 2 measures of health insurance literacy, but the difference in MA enrollment was smaller (33.6% for individuals who reported having information to make an informed comparison vs 29.2% for those who did not, and 33.8% for those who reported that it was easy to review and compare coverage options vs 30.9% for those who did not). Full regression results are presented in eTable 1 in the Supplement. Findings were similar after the inverse probability of treatment weights was applied (eTables 2 and 3 in the Supplement).

**Figure.**
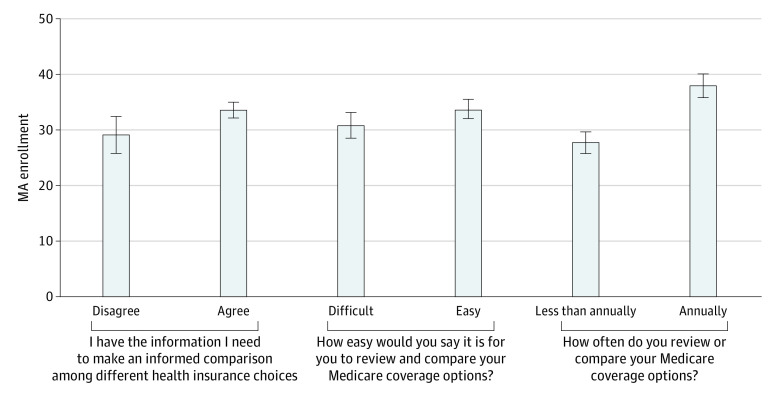
Adjusted Rates of Enrollment in Medicare Advantage Among Medicare Enrollees by Health Insurance Literacy Rates and 95% CIs (represented by the whiskers) were estimated with a linear probability model while controlling for individual-level characteristics (age, sex, race and ethnicity, education, income, Medicare and Medicaid dual eligibility, marital status, self-reported comorbidities, general perceived health status, and limitations of activities of daily living, as well as baseline year), county-level MA plan offerings (total number of MA plans overall and the number of MA plans by star rating, plan type, monthly plan premium, and maximum out-of-pocket limit), and each measure of health insurance literacy. We also included county-fixed associations. Using the predictive marginal associations at representative values estimated from the model, we estimated the adjusted mean values of MA enrollment by health insurance literacy. All estimates were adjusted for survey weighting and design. Each measure of health insurance literacy was asked with a 4-point scale. We categorized responses into 2 levels: disagree (completely or somewhat disagree) vs agree (completely or somewhat agree), difficult (very or somewhat difficult) vs easy (very or somewhat easy), and less than annually (never, rarely, or once every few years) vs annually (at least once every year). MA indicates Medicare Advantage.

Individuals who reviewed or compared coverage options annually were more likely to enroll in plans with 4 to 4.5 stars and in unrated plans (by 4.6 percentage points [95% CI, 0.1-9.2 percentage points] and 2.5 percentage points [95% CI, 0.9-4.1 percentage points], respectively) than those who did not, but were less likely to enroll in plans with 5 stars (by 3.8 percentage points [95% CI, –5.8 to –1.9 percentage points]) (Table 2). Also, enrollment in plans with monthly premiums of $1 to $50 was 4.8 percentage points (95% CI, 0.6-9.0 percentage points) higher among individuals who reviewed or compared coverage options annually than among those who did not. However, no differential pattern was found in enrollment in plans with no monthly premium or monthly premiums higher than $50. Compared with individuals who reviewed or compared coverage options less than annually, those who did so annually were more likely to enroll in plans with a maximum out-of-pocket limit less than $4000 (by 4.7 percentage points [95% CI, 0.3-9.0 percentage points]) but were less likely to enroll in plans with a maximum out-of-pocket limit of $4000 to $5500 (by 6.2 percentage points [95% CI, –10.8 to –1.5 percentage points]). There were no significant differences by plan type and supplemental benefits. We also found limited differences in benefit-specific enrollment in 2 other measures of health insurance literacy.

**Table 2.** Adjusted Rates of Enrollment in a Medicare Advantage Plan With Specific Characteristics by Health Insurance Literacy

Characteristic	Percentage points (95% CI)					
	I have the information I need to make an informed comparison among different health insurance choices		How easy would you say it is for you to review and compare your Medicare coverage options?		How often do you review or compare your Medicare coverage options?	
	Adjusted rate of MA enrollment for “disagree,” %^a^	Difference in adjusted rate of MA enrollment for “agree” from “disagree,” percentage point^b^	Adjusted rate of MA enrollment for “difficult,” %^a^	Difference in adjusted rate of MA enrollment for “easy” from “difficult,” percentage point^b^	Adjusted rate of MA enrollment for “less than annually,” %^a^	Difference in adjusted rate of MA enrollment for “annually” from “less than annually,” percentage point^b^
Star rating						
2-2.5	0.7 (0.6 to 0.7)	0.0 (–0.1 to 0.1)	0.6 (0.6 to 0.6)	0.0 (0.0 to 0.0)	0.6 (0.6 to 0.6)	0.0 (0.0 to 0.0)
3-3.5	32.4 (27.6 to 37.1)	–0.9 (–6.2 to 4.5)	34.0 (30.5 to 37.6)	–3.5 (–8.0 to 0.9)	33.6 (30.2 to 36.9)	–3.3 (–7.7 to 1.1)
4-4.5	53.3 (48.1 to 58.5)	3.7 (–2.0 to 9.4)	55.0 (51.2 to 58.8)	2.2 (–2.5 to 6.9)	54.1 (50.5 to 57.6)	4.6 (0.1 to 9.2)^c^
5	10.1 (7.7 to 12.5)	–3.3 (–5.8 to –0.7)^c^	7.9 (6.2 to 9.7)	–1.0 (–3.0 to 1.0)	9.3 (7.8 to 10.8)	–3.8 (–5.8 to –1.9)^c^
Unrated	3.5 (1.7 to 5.4)	0.4 (–1.7 to 2.6)	2.4 (1.3 to 3.4)	2.4 (0.6 to 4.1)^c^	2.4 (1.4 to 3.5)	2.5 (0.9 to 4.1)^c^
Monthly premium, $						
0	50.8 (45.1 to 56.4)	–3.3 (–9.3 to 2.8)	51.7 (47.6 to 55.7)	–5.4 (–10.3 to –0.5)^c^	48.1 (44.5 to 51.8)	–0.3 (–5.0 to 4.4)
1-50	24.2 (18.8 to 29.5)	–1.9 (–7.7 to 3.9)	20.9 (17.1 to 24.7)	2.4 (–2.3 to 7.2)	19.8 (16.8 to 22.7)	4.8 (0.6 to 9.0)^c^
>51	25.1 (20.5 to 29.7)	5.1 (0.0 to 10.3)	27.4 (23.7 to 31.2)	3.0 (–1.6 to 7.6)	32.1 (28.6 to 35.7)	–4.5 (–9.0 to 0.1)
In-network maximum out-of-pocket limit, $						
<4000	26.9 (22.1 to 31.8)	5.1 (–0.3 to 10.5)	28.9 (25.2 to 32.5)	3.5 (–1.2 to 8.2)	28.6 (25.3 to 31.8)	4.7 (0.3 to 9.0)^c^
4000-5500	28.0 (22.5 to 33.6)	–2.1 (–8.2 to 3.9)	26.1 (22.3 to 29.8)	0.3 (–4.4 to 5.0)	29.8 (26.2 to 33.4)	–6.2 (–10.8 to –1.5)^c^
≥5500	45.0 (39.5 to 50.5)	–3.0 (–9.0 to 3.1)	45.1 (41.3 to 48.9)	–3.8 (–8.6 to 1.0)	41.6 (38.3 to 44.9)	1.5 (–3.0 to 6.1)
Plan type						
HMO	70.1 (65.7 to 74.5)	–1.8 (–6.7 to 3.1)	67.5 (64.3 to 70.7)	1.5 (–2.5 to 5.6)	68.2 (65.1 to 71.3)	0.5 (–3.6 to 4.7)
PPO						
Local	14.0 (10.3 to 17.8)	3.6 (–0.7 to 7.9)	16.5 (13.7 to 19.3)	0.8 (–2.9 to 4.5)	16.7 (13.8 to 19.6)	0.6 (–3.2 to 4.4)
Regional	12.0 (9.0 to 14.9)	–1.4 (–4.8 to 1.9)	12.4 (10.1 to 14.7)	–2.4 (–5.3 to 0.5)	11.7 (9.8 to 13.6)	–1.7 (–4.2 to 0.8)
Other	4.0 (3.1 to 4.8)	–0.3 (–1.3 to 0.6)	3.6 (3.0 to 4.2)	0.0 (–0.6 to 0.7)	3.4 (2.9 to 3.8)	0.6 (–0.1 to 1.3)
Prescription gap coverage						
No	28.7 (21.5 to 35.8)	–6.8 (–14.4 to 0.8)	26.6 (22.1 to 31.2)	–5.5 (–10.9 to –0.1)^c^	24.0 (20.1 to 27.9)	–1.7 (–6.8 to 3.3)
Yes	71.3 (64.2 to 78.5)	6.8 (–0.8 to 14.4)	73.4 (68.8 to 77.9)	5.5 (0.1 to 10.9)^c^	76.0 (72.1 to 79.9)	1.7 (–3.3 to 6.8)
Dental coverage						
No	67.6 (61.6 to 73.6)	–5.6 (–12.8 to 1.5)	62.1 (57.2 to 66.9)	–0.9 (–7.0 to 5.1)	64.1 (59.9 to 68.4)	–4.7 (–10.5 to 1.0)
Yes	32.4 (26.4 to 38.4)	5.6 (–1.5 to 12.8)	37.9 (33.1 to 42.8)	0.9 (–5.1 to 7.0)	35.9 (31.6 to 40.1)	4.7 (–1.0 to 10.5)
Vision coverage						
No	53.9 (47.2 to 60.7)	–1.2 (–7.3 to 4.9)	49.2 (44.4 to 54.1)	–1.2 (–7.3 to 4.9)	46.1 (41.7 to 50.5)	4.2 (–1.8 to 10.1)
Yes	50.8 (45.9 to 55.6)	1.2 (–4.9 to 7.3)	50.8 (45.9 to 55.6)	1.2 (–4.9 to 7.3)	53.9 (49.5 to 58.3)	–4.2 (–10.1 to 1.8)

Abbreviations: HMO, health maintenance organization; MA, Medicare Advantage; PPO, preferred provider organization.

^a^ Values are the adjusted predicted probabilities of enrolling in a plan with a specific characteristic (eg, the average person who “disagrees” that they have the information they need to make an informed choice has a 53.3% probability of selecting a plan rated 4 or 4.5 stars) within strata defined by the column variables. The predicted probabilities for each row variable sum to 100% within the strata defined by the column variables. Rates and 95% CIs were estimated with multinomial logit regression while controlling for individual-level characteristics (age, sex, race and ethnicity, education, income, Medicare and Medicaid dual eligibility, marital status, self-reported comorbidities, general perceived health status, limitations in activities of daily living, and baseline year), county-level MA plan offerings (total number of MA plans overall and the number of MA plans by star rating, plan type, monthly plan premium, and maximum out-of-pocket limit), and each measure of health insurance literacy. We also included other 4 variables of plan characteristics (monthly plan premium, in-network maximum out-of-pocket limit, plan type, and provision of supplemental benefits) to adjust for differences in other plan characteristics. Then, we estimated the adjusted mean values of the outcome by health insurance literacy.

^b^ Estimated differences in the adjusted mean values by health insurance literacy.

^c^ Statistically significant differences (*P* < .05).

Overall, MA enrollees were marginally sensitive to monthly premiums and maximum out-of-pocket limit regardless of health insurance literacy (Table 3). For all 3 measures of health insurance literacy, a $100 increase in monthly premium or a $1000 increase in maximum out-of-pocket limit was associated with a decrease in the likelihood of enrolling in a given plan by approximately 1 percentage point (−0.84 percentage point for individuals who had the information they needed to make an informed comparison among different health insurance choices, −0.84 percentage point for those who reported that it was easy to review and compare coverage options, and −1.12 or −1.13 percentage points for those who reviewed or compared coverage options annually). There were notable findings by whether to review or compare coverage options annually, although the magnitude of the responsiveness was marginal. First, individuals who reviewed or compared coverage options annually were more responsive to changes in monthly premium and maximum out-of-pocket limit than those who did not. Second, there was a differential enrollment pattern by star rating. For individuals who reviewed or compared coverage options annually, a change in rating from 2-2.5 stars to 3-3.5 stars was associated with an increase of 0.12 percentage point in the likelihood of enrolling in a given plan. However, a change in rating from 4 to 4.5 stars to 5 stars was associated with a decrease of 0.31 percentage point in the likelihood of enrolling in a given plan. However, an opposite trend was found among individuals who reviewed or compared coverage options less than annually. Full regression results are presented in eTable 4 in the Supplement.

**Table 3.** Marginal Changes in Benefit-Specific Enrollment in Medicare Advantage (MA) Among MA Enrollees by Health Insurance Literacy

Characteristic	Marginal association, percentage point^a^					
	I have the information I need to make an informed comparison among different health insurance choices		How easy would you say it is for you to review and compare your Medicare coverage options?		How often do you review or compare your Medicare coverage options?	
	Disagree	Agree	Difficult	Easy	Less than annually	Annually
Change in star rating						
From 2-2.5 to 3-3.5	–0.01	0.00	0.01	–0.01	–0.16	0.12
From 3-3.5 to 4-4.5	0.00	0.00	0.00	0.00	0.00	0.00
From 4-4.5 to 5	0.00	0.00	–0.01	0.01	0.41	–0.31
$100 Increase in premium	–1.04	–0.84	–1.06	–0.84	–0.85^b^	–1.12^b^
$1000 Increase in in-network maximum out-of-pocket limit	–1.04	–0.84	–1.06	–0.84	–0.84	–1.13
Plan type						
From HMO to local PPO	0.00	0.00	0.01	–0.01	–0.09	0.07
From HMO to regional PPO	0.00	0.00	–0.01	0.01	0.05	–0.04
From HMO to other	0.00	0.00	0.01	–0.01	–0.02	0.02
Supplemental benefits						
From none to prescription gap coverage	0.00	0.00	–0.01	0.01	–0.04	0.03
From none to dental coverage	0.00	0.00	0.00	0.00	–0.01	0.01
From none to vision coverage	0.00	0.00	0.00	0.00	0.01	–0.01

Abbreviations: HMO, health maintenance organization; PPO, preferred provider organization.

^a^ The marginal associations were estimated with conditional logit regression while controlling for plan characteristics (star rating, monthly premium, in-network maximum out-of-pocket limit, plan type, and supplemental benefits), as well as their interaction terms with each measure of health insurance literacy. Then, the differential associations were estimated between an MA plan’s characteristic and the probability that an enrollee would select that MA plan. These results were presented as the marginal association between a 1-unit change in a characteristic and the probability of selecting an MA plan. For example, an increase in plans’ rating from 4 to 4.5 stars to 5 stars was associated with an increase of 0.41 percentage point in the likelihood of enrolling in a given plan among MA enrollees who reviewed or compared Medicare coverage options less than annually and was associated with a decrease of 0.31 percentage point in the likelihood of enrolling in a given plan among MA enrollees who reviewed or compared Medicare coverage options annually.

^b^ *P* < .05.

High health insurance literacy tended to be particularly low among individuals with poor health or low socioeconomic status (Table 4). The likelihood of reviewing or comparing coverage options annually was higher among women than men (odds ratio [OR], 1.16; 95% CI, 1.01-1.34), those with high school completion (OR, 1.26; 95% CI, 1.04-1.54) or at least a college degree (OR, 1.32; 95% CI, 1.07-1.62) than those with less than a high school education, and those who were married than those who were not (OR, 1.18; 95% CI, 1.02-1.38), but lower among those who were older than 80 years than those younger than 65 years (OR, 0.70; 95% CI, 0.55-0.89), for those with Medicare and Medicaid dual eligibility than for those without it (OR, 0.79; 95% CI, 0.63-0.99), and those with at least 3 limitations in activities of daily living than those without any (OR, 0.68; 95% CI, 0.55-0.84).

**Table 4.** Characteristics Associated With Health Insurance Literacy Among Medicare Enrollees

Variable	OR (95% CI)^a^		
	I have the information I need to make an informed comparison among different health insurance choices (agree)	How easy would you say it is for you to review and compare your Medicare coverage options? (easy)	How often do you review or compare your Medicare coverage options? (annually)
Age, y			
<65	1 [Reference]	1 [Reference]	1 [Reference]
65-70	1.36 (0.99-1.86)	0.99 (0.75-1.29)	0.93 (0.72-1.21)
71-75	1.05 (0.79-1.40)	0.89 (0.69-1.16)	0.93 (0.72-1.19)
76-80	1.11 (0.82-1.50)	0.90 (0.69-1.17)	0.87 (0.68-1.13)
>80	1.30 (0.98-1.74)	0.98 (0.76-1.26)	0.70 (0.55-0.89)^b^
Men			
Women	1.10 (0.93-1.31)	0.88 (0.76-1.02)	1.16 (1.01-1.34)^c^
Race and ethnicity			
Hispanic	1.11 (0.76-1.61)	1.04 (0.78-1.40)	0.91 (0.68-1.21)
Non-Hispanic			
Asian	0.52 (0.24-1.14)	1.44 (0.67-3.11)	0.66 (0.36-1.21)
Black	1.08 (0.83-1.41)	1.29 (1.03-1.63)^d^	1.13 (0.90-1.42)
White	1 [Reference]	1 [Reference]	1 [Reference]
Other^e^	1.19 (0.77-1.85)	1.21 (0.85-1.73)	1.23 (0.85-1.79)
Education			
High school	1 [Reference]	1 [Reference]	1 [Reference]
High school completion	1.08 (0.84-1.37)	1.07 (0.89-1.29)	1.26 (1.04-1.54)^b^
≥College degree	0.98 (0.76-1.27)	1.12 (0.91-1.38)	1.32 (1.07-1.62)^b^
Income, $			
<25 000	1 [Reference]	1 [Reference]	1 [Reference]
25 000-40 000	1.13 (0.90-1.42)	1.26 (1.02-1.55)^d^	0.89 (0.75-1.07)
>40 000	1.16 (0.90-1.50)	1.11 (0.89-1.37)	0.92 (0.75-1.12)
Medicare-Medicaid dual eligibility	1.21 (0.92-1.59)	1.02 (0.87-1.19)	0.79 (0.63-0.99)^d^
Married	1.31 (1.08-1.60)^b^	1.03 (0.82-1.30)	1.18 (1.02-1.38)^d^
Self-reported comorbidity			
Atherosclerosis	1.01 (0.75-1.35)	0.95 (0.75-1.20)	1.08 (0.85-1.36)
Hypertension	0.88 (0.72-1.06)	0.96 (0.82-1.12)	0.99 (0.85-1.15)
Myocardial infarction	0.76 (0.58-1.00)	0.84 (0.67-1.05)	0.94 (0.75-1.18)
Stroke	0.82 (0.64-1.05)	0.88 (0.71-1.08)	0.95 (0.77-1.17)
Coronary heart disease	1.46 (1.10-1.93)^b^	1.36 (1.07-1.71)^d^	1.57 (1.25-1.97)^c^
Cancer	1.08 (0.90-1.29)	1.10 (0.95-1.27)	0.92 (0.80-1.06)
Rheumatoid arthritis	0.98 (0.79-1.22)	1.04 (0.86-1.25)	1.16 (0.95-1.41)
Diabetes	0.97 (0.79-1.19)	1.01 (0.85-1.19)	1.07 (0.90-1.26)
Alzheimer disease and related dementias	0.97 (0.65-1.43)	0.76 (0.54-1.08)	0.86 (0.60-1.23)
Mental illness	0.72 (0.60-0.86)^c^	0.74 (0.63-0.88)^c^	0.93 (0.78-1.09)
General perceived health status			
Poor	1 [Reference]	1 [Reference]	1 [Reference]
Good	1.09 (0.87-1.37)	1.26 (1.04-1.53)^d^	1.10 (0.91-1.33)
No. of ADL limitations			
0	1 [Reference]	1 [Reference]	1 [Reference]
1-2	0.59 (0.48-0.73)^c^	0.62 (0.52-0.74)^c^	0.92 (0.78-1.08)
≥3	0.41 (0.32-0.53) ^c^	0.42 (0.34-0.52)^c^	0.68 (0.55-0.84)^c^
No. of MA plans	0.95 (0.85-1.05)	0.99 (0.91-1.08)	1.00 (0.93-1.08)
No. of MA plans by star rating			
2-2.5	1.06 (0.89-1.26)	1.01 (0.88-1.16)	0.97 (0.85-1.11)
3-3.5	1.04 (0.94-1.16)	1.00 (0.93-1.08)	0.97 (0.91-1.05)
4-4.5	1.04 (0.94-1.16)	1.00 (0.93-1.08)	0.95 (0.89-1.03)
5	1 [Reference]	1 [Reference]	1 [Reference]
No. of MA plans by plan type			
HMO	1.02 (0.95-1.09)	1.02 (0.96-1.08)	1.05 (0.99-1.11)
PPO			
Local	0.98 (0.92-1.05)	0.97 (0.92-1.03)	1.03 (0.98-1.09)
Regional	1.11 (0.97-1.26)	1.05 (0.94-1.17)	1.19 (1.06-1.32)^b^
Other	1 [Reference]	1 [Reference]	1 [Reference]
No. of MA plans by monthly plan premium, $			
0	0.97 (0.91-1.02)	0.99 (0.94-1.04)	0.97 (0.92-1.01)
>0-50	1.00 (0.93-1.08)	0.98 (0.92-1.04)	0.96 (0.91-1.02)
>50-100	0.94 (0.86-1.03)	1.01 (0.94-1.09)	1.01 (0.94-1.09)
>100	1 [Reference]	1 [Reference]	1 [Reference]
No. of MA plans by in-network maximum out-of-pocket limit, $			
0-3000	1.00 (0.92-1.09)	0.99 (0.93-1.07)	1.08 (1.01-1.15)^d^
>3000-4500	1.02 (0.98-1.06)	0.99 (0.97-1.02)	1.02 (0.99-1.06)
>4500-6000	1.08 (1.02-1.14)	1.01 (0.97-1.06)	1.03 (0.99-1.08)
>6000	1 [Reference]	1 [Reference]	1 [Reference]
Baseline year			
2015	1 [Reference]	1 [Reference]	1 [Reference]
2016	1.15 (0.97-1.37)	1.10 (0.95-1.27)	1.01 (0.88-1.16)

Abbreviations: ADL, activities of daily living; HMO, health maintenance organization; MA, Medicare Advantage; OR, odds ratio; PPO, preferred provider organization.

^a^ For each measure of health insurance literacy, logistic regression was conducted. We coded 1 for “agree” for the statement “I have the information I need to make an informed comparison among different health insurance choices,” “easy” for the question “How easy would you say it is for you to review and compare your Medicare coverage options?” and “annually” for the question “How often do you review or compare your Medicare coverage options?”

^b^ *P* < .005.

^c^ *P* < .001.

^d^ *P* < .05.

^e^ Other included single races not of Hispanic origin (including American Indian or Alaska Native and Native Hawaiian) or individuals who were 2 or more races.

## Discussion

Our study presents 3 key findings. First, individuals with high health insurance literacy were more likely to choose MA than those with low health insurance literacy, consistent across all 3 measures of health insurance literacy. Second, annual review or comparison of coverage options had a stronger association with plan characteristics than the associations between either access to or understanding of plan information and plan characteristics. Specifically, individuals who reviewed or compared coverage options annually were more likely to enroll in low-cost or high-rated MA plans. Third, overall health insurance literacy was particularly low among individuals with low socioeconomic status.

Our findings suggest that health insurance literacy is an important factor in enrollment choice by enabling beneficiaries to more closely align their health insurance needs and preferences. This association may be more relevant to MA because of its low premiums, expanded benefits, and more coordinated care.^14,19^ This finding also indicates that TM enrollment decisions may be attributable to lack of health insurance literacy, because approximately one-half of Medicare beneficiaries did not know about MA during an open enrollment period.^20^ This may be amplified by a status quo bias.^12^ However, some beneficiaries may choose TM owing to preferences such as freedom of provider choice or eligibility for Medigap purchase.

Individuals who reviewed or compared coverage options annually were more likely to enroll in lower-premium plans or high-rated plans than those who did not. First, prior research showed that individuals with sufficient information about plan options enrolled in less costly plans,^7^ which may align with our finding that those who more frequently reviewed their coverage options more often selected lower-premium plans. We also found that individuals who reviewed or compared coverage options annually had higher enrollment in 4- to 4.5-star plans than those who did not, but lower enrollment in 5-star plans. This finding suggests that MA enrollees prefer highly rated plans. Because they must balance preferences across multiple plan characteristics, however, the best-rated plan may not always represent the preferred choice.^21^ One explanation for this finding may be that MA enrollees recognized that a higher star rating was associated with improved patient outcomes, but the magnitude of the improvement was marginal.^22^

Our findings suggest disparities in health insurance literacy by socioeconomic status, consistent with prior research.^23^ There may be a mediating role of socioeconomic status on health insurance literacy. Because individuals who might have potentially improved coverage choices were the least likely to review or compare coverage options annually, programs targeted to them may help to enhance decision-making quality for disadvantaged populations, possibly reducing disparities in enrollment decisions. Because we did not specifically examine each of the associations, however, further research is warranted to explore the underlying mechanisms for each socioeconomic factor. We also found that health insurance literacy varied by disease, which may come from disease-specific variations in insurance coverage, possibly leading to variations in health insurance literacy.

Our findings provide policy implications for enrollment decisions. Many resources are offered by CMS for beneficiaries to better understand and navigate the Medicare program. Overall, seniors tend to prefer person-to-person interaction over existing information resources from CMS,^24^ probably because of a combination of low technological skills and health insurance literacy. To improve health insurance literacy and help beneficiaries make an informed choice, policy makers need to consider developing more decision support tools. Because beneficiaries have various preferences and needs, the tools need to account for individual-specific characteristics. Specifically, tools can be designed to personalize choice framing and recommendations based on an individual’s specific characteristics. Prior research found that consumers with personalized information were more likely to switch plans and to choose a low-cost plan than those without it.^25^

### Limitations

Our study has several limitations. First, an individual’s plan choice may be affected by additional aspects of plan benefits. However, evidence suggests that the role of other benefits is limited in enrollment decisions.^18^ Second, estimates of the association between health insurance literacy and TM vs MA enrollment may be biased because of differences in individual-level characteristics that influence both literacy and enrollment. Third, there may be selective enrollment within MA by plan characteristics. Healthy enrollees may enroll more in high-rated plans than sick enrollees. Fourth, we could not account for all possible factors that may affect insurance coverage. Because our data did not allow us to determine the extent to which beneficiaries were making optimal choices, our findings should be interpreted with caution. Fifth, our analysis is associational, and our results do not necessarily have a causal interpretation.

## Conclusions

In this cross-sectional study, higher health insurance literacy—particularly, annual review and comparison of coverage choices—was associated with higher MA enrollment and choice of a particular MA plan. Policy makers should develop programs to make health insurance information accessible and understandable across diverse and older adult populations and to encourage annual review and comparison of coverage options.

## Notes

Supplement. **eTable 1.** Results From Linear Probability Models of Enrollment in Medicare Advantage (MA) **eTable 2.** Sample Characteristics After Applying Inverse Probability of Treatment Weighting **eTable 3.** Adjusted Rates of Enrollment in Medicare Advantage (MA) With and Without Inverse Probability of Treatment Weighting (IPTW) **eTable 4.** Results From Conditional Logistic Regression of Enrollment in Medicare Advantage (MA)
