# Supplementary material for: Association of Health Insurance Literacy With Enrollment in Traditional Medicare, Medicare Advantage, and Plan Characteristics Within Medicare Advantage
Source: JAMA Netw Open. 2022 Feb 3;5(2):e2146792. doi: 10.1001/jamanetworkopen.2021.46792 (PMC8814909; doi:10.1001/jamanetworkopen.2021.46792)
Supplement: Supplement. — eTable 1. Results From Linear Probability Models of Enrollment in Medicare Advantage (MA) eTable 2. Sample Characteristics After Applying Inverse Probability of Treatment Weighting eTable 3. Adjusted Rates of Enrollment in Medicare Advantage (MA) With and Without Inverse Probability of Treatment Weighting (IPTW) eTable 4. Results From Conditional Logistic Regression of Enrollment in Medicare Advantage (MA) [file jamanetwopen-e2146792-s001.pdf]

Supplemental Online Content

Park S, Langellier BA, Meyers DJ. Association of health insurance literacy with enrollment in traditional Medicare, Medicare Advantage, and plan characteristics within Medicare Advantage. *JAMA Netw Open.* 2022;5(2):e2146792. doi:10.1001/jamanetworkopen.2021.46792

eTable 1. Results From Linear Probability Models of Enrollment in Medicare Advantage (MA)

eTable 2. Sample Characteristics After Applying Inverse Probability of Treatment Weighting

eTable 3. Adjusted Rates of Enrollment in Medicare Advantage (MA) With and Without Inverse Probability of Treatment Weighting (IPTW)

eTable 4. Results From Conditional Logistic Regression of Enrollment in Medicare Advantage (MA)

This supplemental material has been provided by the authors to give readers additional information about their work.

© 2022 Park S et al. *JAMA Network Open.*

eTable 1. Results From Linear Probability Models of Enrollment in Medicare Advantage (MA)

	MA enrollment, OR (95% CI)		
Variable	Model 1	Model 2	Model 3
I have the information I need to make an informed comparison among different health insurance choices			
Disagree	REF		
Agree	**1.32 (1.09 to 1.6)**		
How easy would you say it is for you to review and compare your Medicare coverage options?			
Difficult		REF	
Easy		**1.22 (1.04 to 1.43)**	
How often do you review or compare your Medicare coverage options?			
Less than annually			REF
Annually			**1.72 (1.47 to 2.01)**
Age			
<65	REF	REF	REF
65-70	1.24 (0.91 to 1.67)	1.26 (0.93 to 1.7)	1.27 (0.93 to 1.72)
71-75	**1.33 (1 to 1.78)**	**1.35 (1.01 to 1.8)**	**1.36 (1.01 to 1.82)**
76-80	1.18 (0.88 to 1.6)	1.2 (0.89 to 1.62)	1.22 (0.9 to 1.65)
>80	1.15 (0.87 to 1.54)	1.17 (0.88 to 1.56)	1.23 (0.92 to 1.64)
Female	1.12 (0.96 to 1.32)	1.13 (0.96 to 1.33)	1.11 (0.94 to 1.31)
Race/ethnicity			
Non-Hispanic White	REF	REF	REF
Non-Hispanic Black	0.99 (0.77 to 1.29)	0.99 (0.76 to 1.28)	0.99 (0.76 to 1.28)
Non-Hispanic Asian	0.51 (0.24 to 1.09)	0.49 (0.22 to 1.06)	0.53 (0.25 to 1.13)
Hispanic	**1.37 (1.02 to 1.85)**	**1.37 (1.02 to 1.84)**	**1.4 (1.03 to 1.9)**
Others	**1.54 (1 to 2.38)**	**1.54 (1 to 2.37)**	**1.52 (1 to 2.3)**
Education			
Less than high school	REF	REF	REF
High school completion	1 (0.81 to 1.24)	0.99 (0.8 to 1.23)	0.97 (0.78 to 1.21)
College degree and greater	**0.78 (0.62 to 0.98)**	**0.78 (0.62 to 0.98)**	**0.75 (0.6 to 0.95)**
Income			
Less than $25,000	REF	REF	REF
$25,000-$40,000	**0.78 (0.64 to 0.95)**	**0.79 (0.65 to 0.96)**	**0.8 (0.65 to 0.97)**

© 2022 Park S et al. *JAMA Network Open.*

More than $40,000	**0.47 (0.38 to 0.59)**	**0.47 (0.38 to 0.59)**	**0.47 (0.38 to 0.59)**
Dual eligibility	**0.46 (0.35 to 0.61)**	**0.47 (0.35 to 0.62)**	**0.48 (0.36 to 0.64)**
Married	**1.32 (1.11 to 1.56)**	**1.33 (1.12 to 1.58)**	**1.31 (1.1 to 1.55)**
Self-reported comorbidity			
Hardening of arteries	1.05 (0.8 to 1.38)	1.05 (0.8 to 1.37)	1.04 (0.79 to 1.36)
Hypertension	0.9 (0.75 to 1.07)	0.9 (0.75 to 1.07)	0.89 (0.75 to 1.07)
Myocardial infarction	**1.33 (1.01 to 1.74)**	**1.32 (1.01 to 1.73)**	**1.33 (1.02 to 1.75)**
Stroke	1.25 (0.98 to 1.6)	1.25 (0.97 to 1.59)	1.25 (0.97 to 1.6)
Coronary heart disease	1.19 (0.91 to 1.55)	1.19 (0.92 to 1.55)	1.14 (0.87 to 1.48)
Cancer	**0.84 (0.71 to 0.98)**	**0.83 (0.71 to 0.98)**	**0.84 (0.72 to 0.99)**
Rheumatoid arthritis	0.86 (0.69 to 1.06)	0.86 (0.69 to 1.06)	0.84 (0.68 to 1.04)
Diabetes	1.07 (0.89 to 1.28)	1.07 (0.89 to 1.28)	1.06 (0.88 to 1.27)
Alzheimer's disease and related dementias	0.99 (0.66 to 1.51)	1 (0.66 to 1.52)	1 (0.67 to 1.51)
Mental illness	0.97 (0.81 to 1.17)	0.97 (0.81 to 1.17)	0.97 (0.81 to 1.17)
General perceived health status			
Good	REF	REF	REF
Poor	1.17 (0.94 to 1.46)	1.16 (0.94 to 1.45)	1.16 (0.93 to 1.45)
Number of ADL limitations			
0	REF	REF	REF
1-2	0.94 (0.78 to 1.14)	0.94 (0.78 to 1.14)	0.94 (0.78 to 1.13)
More than 3	0.86 (0.68 to 1.1)	0.86 (0.68 to 1.1)	0.87 (0.68 to 1.11)
Number of MA plans	0.99 (0.91 to 1.08)	0.99 (0.91 to 1.08)	0.99 (0.91 to 1.08)
Number of MA plans by star rating			
2-2.5 stars	0.99 (0.86 to 1.14)	1 (0.86 to 1.15)	1 (0.87 to 1.15)
3-3.5 stars	0.99 (0.92 to 1.07)	0.99 (0.92 to 1.07)	1 (0.92 to 1.08)
4-4.5 stars	1.04 (0.96 to 1.12)	1.04 (0.96 to 1.13)	1.05 (0.97 to 1.14)
5 stars	REF	REF	REF
Nubmer of MA plans by plan type			
HMO	1.01 (0.95 to 1.07)	1.01 (0.95 to 1.07)	1 (0.95 to 1.06)
Local PPO	1.03 (0.97 to 1.09)	1.03 (0.97 to 1.09)	1.02 (0.97 to 1.08)
Regional PPO	0.98 (0.87 to 1.1)	0.98 (0.87 to 1.1)	0.96 (0.85 to 1.08)
Other	REF	REF	REF
Nubmer of MA plans by monthly plan premium			
$0	**1.06 (1.01 to 1.11)**	**1.06 (1 to 1.11)**	**1.06 (1.01 to 1.11)**
>$0-$50	0.99 (0.93 to 1.05)	0.99 (0.93 to 1.06)	0.99 (0.93 to 1.06)
>$50-$100	0.97 (0.89 to 1.05)	0.97 (0.89 to 1.05)	0.96 (0.88 to 1.05)
>$100	REF	REF	REF

© 2022 Park et al. *JAMA Network Open.*

Nubmer of MA plans by maximum out-of-pocket limit			
$0-$3,000	**1.19 (1.11 to 1.28)**	**1.19 (1.11 to 1.28)**	**1.18 (1.1 to 1.27)**
>$3,000-$4,500	0.98 (0.95 to 1.01)	0.98 (0.95 to 1.01)	0.97 (0.94 to 1)
>$4,500-$6,000	**1.12 (1.07 to 1.18)**	**1.12 (1.07 to 1.18)**	**1.12 (1.07 to 1.18)**
>$6,000	REF	REF	REF
Baseline year			
2015	REF	REF	REF
2016	0.98 (0.84 to 1.16)	0.99 (0.84 to 1.16)	0.99 (0.84 to 1.16)

Abbreviation: MA, Medicare Advantage; ADL, activities of daily living; HMO, health maintenance organization; PPO, preferred provider organization.

Bold indicates statistically significant differences ($p < 0.05$).

© 2022 Park et al. *JAMA Network Open.*

eTable 2. Sample Characteristics After Applying Inverse Probability of Treatment Weighting

Characteristic	TM enrollees	MA enrollees
Individual-level characteristics of demographic, socioeconomic, and health status		
Age, %		
<65	15.6	14.9
65-70	27.7	29.0
71-75	21.9	23.8
76-80	15.7	15.4
>80	19.1	16.8
Female, %	55.9	56.1
Race/ethnicity, %		
Non-Hispanic White	79.0	78.1
Non-Hispanic Black	11.0	11.4
Non-Hispanic Asian	1.8	2.1
Hispanic	7.3	7.2
Others	3.5	3.4
Education, %		
Less than high school	13.6	13.8
High school completion	36.3	36.1
College degree and greater	49.8	49.9
Income, %		
Less than $25,000	38.7	38.8
$25,000-$40,000	28.8	28.7
More than $40,000	32.6	32.5
Dual eligibility, %	13.3	13.8
Married, %	54.7	54.5
Self-reported comorbidity, %		
Hardening of arteries	10.4	10.1
Hypertension	67.1	66.9
Myocardial infarction	11.7	11.9
Stroke	10.3	10.3
Coronary heart disease	11.1	10.9
Cancer	33.7	33.7
Rheumatoid arthritis	16.6	17.5
Diabetes	31.5	32.6
Alzheimer's disease and related dementias	2.9	3.0

© 2022 Park et al. *JAMA Network Open.*

Mental illness	30.2	29.6
General perceived health status, %		
Good	78.6	78.6
Poor	21.4	21.4
Number of ADL limitations, %		
0	59.9	60.0
1-2	22.9	22.6
More than 3	17.2	17.5
County-level characteristics of MA plan offering		
Total number of MA plans, mean (SD)	12.9 (8.0)	13.0 (7.1)
Star rating, mean (SD)		
2-2.5 stars	0.2 (0.6)	0.2 (0.6)
3-3.5 stars	5.1 (5.3)	5.2 (4.9)
4-4.5 stars	7.1 (5.3)	7.1 (5.1)
5 stars	0.4 (1.1)	0.4 (1.1)
Plan type, mean (SD)		
HMO	7.8 (7.3)	7.9 (6.4)
Local PPO	3.2 (2.8)	3.2 (2.8)
Regional PPO	1.4 (1.2)	1.4 (1.3)
Other	0.5 (1.9)	0.4 (2.0)
Monthly plan premium, mean (SD)		
$0	4.4 (5.3)	4.5 (4.7)
>$0-$50	3.1 (2.7)	3.1 (2.5)
>$50-$100	2.5 (1.7)	2.5 (1.6)
>$100	2.9 (2.8)	2.8 (2.8)
Maximum out-of-pocket limit, mean (SD)		
$0-$3,000	0.7 (1.5)	0.6 (1.5)
>$3,000-$4,500	4.2 (4.2)	4.2 (3.8)
>$4,500-$6,000	2.4 (2.0)	2.4 (2.0)
>$6,000	5.7 (4.6)	5.7 (4.4)

Abbreviation: TM, traditional Medicare; MA, Medicare Advantage; ADL, activities of daily living; HMO, health maintenance organization; PPO, preferred provider organization.

© 2022 Park et al. *JAMA Network Open.*

eTable 3. Adjusted Rates of Enrollment in Medicare Advantage (MA) With and Without Inverse Probability of Treatment Weighting (IPTW)

	Adjusted rates of MA enrollment								
	I have the information I need to make an informed comparison among different health insurance choices			How easy would you say it is for you to review and compare your Medicare coverage options?			How often do you review or compare your Medicare coverage options?		
	Adjusted rate of MA enrollment for "disagree", % (95% CI)	Adjusted rate of MA enrollment for "agree", % (95% CI)	Difference in adjusted rate of MA enrollment for "agree" from "disagree", percentage point (95% CI)	Adjusted rate of MA enrollment for "difficult", % (95% CI)	Adjusted rate of MA enrollment for "easy", % (95% CI)	Difference in adjusted rate of MA enrollment for "easy" from "difficult", percentage point (95% CI)	Adjusted rate of MA enrollment for "less than annually", % (95% CI)	Adjusted rate of MA enrollment for "annually", % (95% CI)	Difference in adjusted rate of MA enrollment for "annually" from "less than annually", percentage point (95% CI)
Without IPTW matching	29.2 (26.0 to 32.5)	33.6 (32.1 to 35.0)	4.3 (0.7 to 8.0)	30.9 (28.5 to 33.2)	33.8 (32.1 to 35.5)	3.0 (0.0 to 5.9)	27.8 (25.8 to 29.7)	38.0 (36.0 to 40.1)	10.3 (7.4 to 13.2)
With IPTW matching	46.8 (42.9 to 50.7)	50.5 (48.7 to 52.3)	3.7 (-0.6 to 8.0)	47.8 (45.1 to 50.6)	50.9 (48.9 to 52.9)	3.0 (-0.5 to 6.5)	43.8 (41.4 to 46.1)	55.8 (53.5 to 58.1)	12.0 (8.6 to 15.4)

Abbreviation: MA, Medicare Advantage; IPTW, inverse probability of treatment weighting.

© 2022 Park et al. *JAMA Network Open.*

eTable 4. Results From Conditional Logistic Regression of Enrollment in Medicare Advantage (MA)

	MA enrollment, OR (95% CI)		
Variable	Model 1	Model 2	Model 3
I have the information I need to make an informed comparison among different health insurance choices [Agree]	0.55 (0.12 to 2.47)		
Star rating			
2-2.5 stars	REF		
3-3.5 stars	1.96 (0.59 to 6.44)		
4-4.5 stars	2.82 (0.86 to 9.21)		
5 stars	**7.27 (1.93 to 27.38)**		
Unrated	0.64 (0.16 to 2.56)		
Interaction between star rating and "agree"			
2-2.5 stars	REF		
3-3.5 stars	1.59 (0.4 to 6.39)		
4-4.5 stars	1.69 (0.42 to 6.73)		
5 stars	1.64 (0.36 to 7.55)		
Unrated	0.86 (0.17 to 4.36)		
Monthly premium	0.99 (0.99 to 1)		
Interaction between monthly premium and "agree"	**1 (1 to 1)**		
Maximum out-of-pocket limit	**1 (1 to 1)**		
Interaction between maximum out-of-pocket limit and "agree"	**1 (1 to 1)**		
Plan type			
HMO	REF		
Local PPO	**0.59 (0.41 to 0.85)**		
Regional PPO	0.79 (0.47 to 1.31)		
Other	0.68 (0.21 to 2.22)		
Interaction between plan type and "agree"			
HMO	REF		
Local PPO	1.11 (0.74 to 1.65)		
Regional PPO	1.14 (0.65 to 1.99)		
Other	0.94 (0.27 to 3.34)		
Supplemental benefits			
Prescription gap coverage	**3.06 (2.18 to 4.3)**		
Dental coverage	0.92 (0.44 to 1.9)		
Vision coverage	0.89 (0.4 to 1.99)		
Interaction between supplemental benefits and "agree"			
Prescription gap coverage	1.09 (0.75 to 1.58)		
Dental coverage	0.98 (0.5 to 1.93)		

© 2022 Park et al. *JAMA Network Open.*

Vision coverage	1.04 (0.52 to 2.07)		
How easy would you say it is for you to review and compare your Medicare coverage options? [Easy]		1.32 (0.3 to 5.7)	
Star rating			
2-2.5 stars		REF	
3-3.5 stars		**3.38 (1.05 to 10.87)**	
4-4.5 stars		**4.86 (1.52 to 15.58)**	
5 stars		**11.29 (3.28 to 38.81)**	
Unrated		0.81 (0.22 to 2.98)	
Interaction between star rating and "easy"			
2-2.5 stars		REF	
3-3.5 stars		0.77 (0.19 to 3.02)	
4-4.5 stars		0.82 (0.21 to 3.24)	
5 stars		0.94 (0.22 to 4.04)	
Unrated		0.57 (0.12 to 2.72)	
Monthly premium		0.99 (0.99 to 1)	
Interaction between monthly premium and "easy"		**1 (1 to 1)**	
Maximum out-of-pocket limit		**1 (1 to 1)**	
Interaction between maximum out-of-pocket limit and "easy"		**1 (1 to 1)**	
Plan type			
HMO		REF	
Local PPO		**0.71 (0.56 to 0.91)**	
Regional PPO		0.88 (0.62 to 1.25)	
Other		0.76 (0.37 to 1.56)	
Interaction between plan type and "easy"			
HMO		REF	
Local PPO		0.86 (0.63 to 1.17)	
Regional PPO		0.99 (0.65 to 1.53)	
Other		0.78 (0.32 to 1.91)	
Supplemental benefits			
Prescription gap coverage		**2.98 (2.35 to 3.77)**	
Dental coverage		0.91 (0.54 to 1.53)	
Vision coverage		0.93 (0.46 to 1.87)	
Interaction between supplemental benefits and "easy"			
Prescription gap coverage		1.16 (0.87 to 1.56)	
Dental coverage		0.99 (0.61 to 1.63)	
Vision coverage		0.98 (0.58 to 1.65)	

© 2022 Park et al. *JAMA Network Open.*

How often do you review or compare your Medicare coverage options? [Annually]			0.7 (0.19 to 2.6)
Star rating			
2-2.5 stars			REF
3-3.5 stars			**2.34 (1.01 to 5.4)**
4-4.5 stars			**3.56 (1.55 to 8.19)**
5 stars			**14.41 (5.92 to 35.1)**
Unrated			0.36 (0.12 to 1.04)
Interaction between star rating and "agree"			
2-2.5 stars			REF
3-3.5 stars			1.43 (0.42 to 4.92)
4-4.5 stars			1.42 (0.42 to 4.85)
5 stars			0.55 (0.15 to 2.04)
Unrated			2.12 (0.49 to 9.11)
Monthly premium			1 (0.99 to 1)
Interaction between monthly premium and "agree"			**1 (1 to 1)**
Maximum out-of-pocket limit			**1 (1 to 1)**
Interaction between maximum out-of-pocket limit and "agree"			**1 (1 to 1)**
Plan type			
HMO			REF
Local PPO			**0.55 (0.44 to 0.7)**
Regional PPO			0.82 (0.6 to 1.12)
Other			0.56 (0.31 to 1.04)
Interaction between plan type and "agree"			
HMO			REF
Local PPO			1.3 (0.97 to 1.75)
Regional PPO			1.13 (0.75 to 1.71)
Other			1.21 (0.51 to 2.85)
Supplemental benefits			
Prescription gap coverage			**3.04 (2.46 to 3.75)**
Dental coverage			0.88 (0.54 to 1.43)
Vision coverage			0.93 (0.48 to 1.78)
Interaction between supplemental benefits and "agree"			
Prescription gap coverage			1.15 (0.87 to 1.53)
Dental coverage			1.05 (0.66 to 1.67)
Vision coverage			0.96 (0.59 to 1.56)

Abbreviation: TM, traditional Medicare; MA, Medicare Advantage; ADL, activities of daily living; HMO, health maintenance organization; PPO, preferred provider organization.

© 2022 Park et al. *JAMA Network Open.*

For each measure of health insurance literacy, logistic regression was conducted. We coded 1 for “agree” for the question “I have the information I need to make an informed comparison among different health insurance choices”, “easy” for the question “How easy would you say it is for you to review and compare your Medicare coverage options?”, and “annually” for the question “How often do you review or compare your Medicare coverage options?”

Bold indicates statistically significant differences ($p < 0.05$).

© 2022 Park et al. *JAMA Network Open.*
